# Tower of London Test: A Comparison between Conventional Statistic Approach and Modelling Based on Artificial Neural Network in Differentiating Fronto-Temporal Dementia from Alzheimer’s Disease

**DOI:** 10.3233/BEN-2011-0327

**Published:** 2011-05-23

**Authors:** Massimo Franceschi, Paolo Caffarra, Rita Savarè, Renata Cerutti, Enzo Grossi, The ToL Research Group

**Affiliations:** ^1^Neurology departmentMultimedica Santa MariaCastellanza (Va)Italy; ^2^Neuroscience departmentUniversità di ParmaConsultorio Disturbi CognitiviParmaItaly and University of HullHullUK; ^3^Direzione Medica FarmaBracco SpaMilanoItaly

**Keywords:** Alzheimer’s disease, frontotemporal dementia, Tower of London, neuropsychology, executive functions

## Abstract

The early differentiation of Alzheimer’s disease (AD) from frontotemporal dementia (FTD) may be difficult. The Tower of London (ToL), thought to assess executive functions such as planning and visuo-spatial working memory, could help in this purpose.

Twentytwo Dementia Centers consecutively recruited patients with early FTD or AD. ToL performances of these groups were analyzed using both the conventional statistical approaches and the Artificial Neural Networks (ANNs) modelling.

Ninety-four non aphasic FTD and 160 AD patients were recruited. ToL Accuracy Score (AS) significantly (*p* < 0.05) The use of hidden information contained in the different items of ToL and the non linear processing of the data through ANNs allows a high discrimination between FTD and AD in individual patients. However, the discriminant validity of AS checked by ROC curve analysis, yielded no significant results in terms of sensitivity and specificity (AUC 0.63). The performances of the 12 Success Subscores (SS) together with age, gender and schooling years were entered into advanced ANNs developed by Semeion Institute. The best ANNs were selected and submitted to ROC curves. The nonlinear model was able to discriminate FTD from AD with an average AUC for 7 independent trials of 0.82.

The use of hidden information contained in the different items of ToL and the non linear processing of the data through ANNs allows a high discrimination between FTD and AD in individual patients.

[1]The ToL Research Group: A. Padovani, Clinica Neurologica, Spedali Civili, Brescia; A. Nieddu, Unità di Geriatria, Policlinico Sassarese, Sassari; F. Clerici, Unità di Neurologia, Ospedale L. Sacco, Milano; G. Bottini, Divisione di Neurologia, Ospedale Niguarda, Milano; F. Lamenza, Unità di Geriatria, Ospedale Civile, Rossano Scalo (CS); G. Gambina, Unità di Neurologia, Azienda Ospedaliera, Verona; G. Magnani, Dipartimento di Neurologia, Ospedale S. Raffaele, Milano; R. Perri, Unità di Neurologia, IRCCS Fondazione S. Lucia, Roma; M. Alberoni, Unità di Neurologia Riabilitativa, Don Gnocchi, Milano; M. Gallucci, Unità di Valutazione Alzheimer, Ospedale Ca' Foncello, Treviso; F. Scoppa, Unità di Neurologia e Psichiatria, Policlinico P. Giaccone, Palermo; S. Lorusso, Unità di Neurologia, Ospedale degli Infermi, Rimini; G. Murialdo, Dipartimento Scienze Endocrinologiche e Mediche, San Martino, Genova; A. Cester, Unità di Geriatria, Ospedale di Dolo (VE); A. Stracciari, Unità di Neurologia, Policlinico S.Orsola Malpighi, Bologna; I. Appollonio, Clinica Neurologica, Ospedale S. Gerardo, Monza; C. Mina, Dipartimento di Neurologia, Policlinico Umberto I, Roma; G. Tripi, Unità di Psicogeriatria, Cittadella della Salute, Erice (TP); L.P. De Vreese, Ambulatorio di Psicogeriatria, Distretto di Castelfranco Emilia (MO); R. Monastero, Unità di Neurologia, Policlinico P. Giaccone, Palermo.

ISSN 0953-4180/11/$27.50 © 2011 – IOS Press and the authors. All rights reserved

## 1. Introduction

Alzheimer's disease (AD) is the prevalent type of dementia in the elderly, followed by FTD which is considered the second commonest cause of dementia in persons younger than 65 [18]. There is also evidence that late onset FTD is not uncommon, generating some difficulties to the differential diagnostic process from frontal variant of AD, when language or dysexecutive deficits are prevalent. An early differentiation between these two forms may help choosing a therapeutic approach with cholinesterases inhibitors which are restricted to AD, while for FTD there is no mention for symptomatic or disease modifying therapy. Secondly, both AD and FTD have significant implications for family members and a correct genetic counselling is largely dependent on a correct diagnosis. Lastly, AD and FTD have different natural histories and prognostic features that patients and their caregivers have to face with.

Besides current clinical criteria, mostly descriptive for FTD [26] and AD [23], several neuropsychological [37], behavioural [16], neuroimaging [29] and functional [12,15] tools have been proposed to achieve an early and reliable differentiation.

With important exceptions [14,37], there is general agreement that neuropsychological tests measuring executive functions, are valid instruments to differentiate AD from FTD [20,28].

Executive deficits, traditionally linked to the prefrontal dysfunction, are heterogeneous and difficult to measure with a single cognitive test [32]. The difficulty is partly a consequence of a large variety of functions subserved by frontal lobes, as well as to the definition of executive functions, which includes a number of abilities, such as planning, set shifting, monitoring, impulse control, abstract reasoning, set maintenance and inhibitory control of actions [10].

The Tower of London (ToL) has been derived from the more complex Tower of Hanoi [2] which is one of the classic puzzles, created by French mathematician Eduardo Lucas in 1883, and originally proposed as a valid tool to study visuospatial planning abilities and problem solving [39].

In a previous study [13] using a simplified version of ToL, AD performed worse than normal controls on planning ability. The goal of the present study was to evaluate the sensitivity of ToL to differentiate AD from FTD in a large sample of subjects, comparing two different statistical approaches, namely a classical analysis vs non linear analysis consisting on artificial neural networks.

## 2. Patients and methods

### 2.1. Patients

Twentytwo Dementia Centers from Universities or General Hospitals agreed to participate to the study. Consecutive patients with probable AD or frontal/dysexecutive variant of FTD according to current research criteria [23,26], with mild to moderate cognitive impairment (MMSE unadjusted score $> 18$ [24]) and no clinical evidence of comprehension deficits entered the study. Patients were dwelling in the community and were free from psychotropic drugs; cholinesterase inhibitors were allowed for AD subjects only when they were on stable dose regimen during the last two months before the participation into the study.

Besides the standard neuropsychological battery used by each Center according to the international and Italian guideline for the diagnosis of dementia, further tests were also used encompassing attention, executive functions, visuospatial and constructional abilities and depression, namely numerical matrices [35], semantic and phonological verbal fluencies [27], the Raven's Coloured Progressive Matrices (RCPM) [3], costructive praxia with (CDP) and without planning (CD) elements [6] and a Geriatric Depression Scale (GDS [34]).

### 2.2. Tower of London

A simplified version of the ToL described by Krikorian et al. [19] has been used in this study, consisting of three wooden pegs of different length, mounted on a strip of wood and three balls of the same size, painted on different colours (red, blue and green) placed on each peg. Patients have to arrange the balls on the pegs in order to achieve a new defined configuration from a predetermined initial position. The task consists of twelve problems (lond 1; lond 2; . . . lond 12.) of graded difficulty to be solved in the least number of moves (2–5). A problem is correctly solved when the end state is achieved in the prescribed number of moves. Three trials are allowed for each problem. The score for each problem ranges from 0 to 3 points.

Three different scores are considered:

a) Success score (SS): 1 point is given for the correct response in each problem and 0 for failure; the score ranges from 0 to 12;
b) Complexity score (CS): the percentage of variation in "difficult" tasks (number of success in items 9–12 requiring up to five moves) in comparison to "easy" tasks (number of success in items 1–4 requiring two or three moves); c) Accuracy score (AS) or total score: three trials are allowed for each problem; 3 points are given for the successful solution on the first trial, 2 points on the second and 1 point on the third trial. A score of zero is given if all three trials are failed. The maximum possible score is 36.

Success Score is a measure of global planning efficiency. The Complexity Score quantifies the ability to cope with more and more complex tasks. The Accuracy Score measures the number of wrong solutions and/or the number of violations of the defined rules (e.g., picking up more than one ball at a time), giving an index of accuracy of the planning.

The original paper by Krikorian also mentioned the possibility of measuring latency and execution times. On the basis of our previous experience [13], we decided to avoid any time measurement, allowing to the patients to complete the task without any time constraint.

Overall, the test took approximately 20 minutes to be admnisitered.

In order to obtain a better homogeneity of the results, a training session for each neuropsychologist involved in the test administration, was established during a dedicated meeting before the study.

### *2.3. Statistical analysis*

Two different ways of analysis were conducted, the first using a classical approach, consisting of independent t test to compare the demographic profile and MMSE global score of patients and controls and Wilcoxon's test to compare AS and CS.

The discriminant validity of AS between FTD and AD patients was checked by ROC curve analysis. Correlation between AS and neuropsychological battery was assessed by Spearman's rank correlation test. All statistical tests, corrected for multiple comparisons, were two tailed and a value of 0.05 or below was accepted as indicator of significance.

Since in an early paper [13] SS and AS did not differ significantly, only AS is presented in this analysis to ascertain the between-group differences and the correlation with the remaining neuropsychological tests.

Normative data for the ToL were collected in a large sample of healthy individuals in another study (paper in preparation). The results showed significant effects of age, sex and education on the individual scores. For this reason adjusted scores were used in our analysis.

Statistical analysis was performed using SAS 8.2.

### *2.4. Methods involving artificial neural networks (ANNs)*

The second statistical approach was based on a non linear analysis by means of ANNs.

ANNs are computer algorithms inspired by the highly interactive processing of the human brain. Like the brain, they can recognize patterns, manage data and learn. When exposed to a complex data set, they recognize the underlying mechanisms of time series and outcomes, thus identifying complex interactions among input data, and recognising hidden relations which usually are not apparent when traditional statistical approaches are used. They are particularly suited for solving problems of the non linear type, being able to reconstruct the approximate rules that put a certain set of data – which describes the problem being considered – with a set of data which provides the solution.

These decision-support systems, based on novel mathematical laws made their entry into medicine several years ago [43], and efforts to improve predictive and prognostic performance of these systems have led to their application tools for clinical decision-making [8,9,45]. ANN are highly flexible computerized mathematical models for understanding and predicting complex and chaotic dynamics in complex biological systems, and have been effectively used to solve non-linear problems related to diagnostic or prognostic queries [4,8]. Thus, ANN would appear to be a promising tool for clinical decision-making and have been applied in various areas of Alzheimer research [11,21, 31]. Artificial Neural Networks (ANNs) are adaptive models for the analysis of data which are inspired by the functioning processes of the human brain. They are systems which are able to modify their internal structure in relation to a function objective.

In this study, supervised ANNs networks were employed, where the output desired was already defined. The input variables to feed AANs were represented by the following variables: gender (male; female) age (years); schooling (education years); performance to each TOL items (lond 1; lond 2. . . lond 12) plus total score (lond tot) These variables operated as independent variables. Output variables, operating as dependant variables, were FDT and AD diagnosis.

The ANNs employed in this analysis had the following architecture:

– the input vector had number of nodes equal to the number of independent variables (17);
– the output vector had two nodes corresponding to the two different diagnoses FTD vs AD;

– 1 layer of hidden units.

Supervised ANNs along a sufficient number of recursive equations application (at least 1000 times) calculated an error function measuring the distance between the desired fixed output (target) and their own output, adjusting during this training process the values of the numerical weights of connections among input nodes, hidden layer nodes, and output nodes to minimize the result of the error function.

The learning constraint of the supervised ANNs aims to make the ANN output coincide with the predefined target, i.e. the actual diagnosis of each patient. The general form of these ANNs is: y = f(x,w*), where w* constitutes the set of parameters which best approximate the function. The ANNs used in the study are characterized by the law of learning and topology. The laws of learning identify equations which translate the ANNs inputs into outputs, and rules by which the weights are modified to minimize the error or the internal energy of the ANNs.

In this study we have used as standard model the The Back Propagation standard (BP-FF) which belongs to a very large family of ANNs defined by different interconnected layers of nodes characterized by a non linear function, which can be differentiated and is limited, that has a linear combination of the activations coming from the previous layer in input. Generally the function in question is of the sigmoidal type. The fundamental equation that characterizes the activation of a single node and therefore, the transfer of the signal from one layer to another is:

$$x_j^{[s]} = f\left(\sum_{i=0}^{n} w_{ji}^{[s]} \cdot x_i^{[s-1]}\right)$$

The validation protocol is a fundamental procedure to verify the models' ability to generalize the results reached in the testing phase. Among the different protocols reported in literature, the selected model is the protocol with the greatest generalization ability on data unknown to the model itself.

The procedural steps in developing the validation protocol rely on the following:

1. subdividing the dataset randomly into two subsamples: the first called Training Set, and the second, called Testing Set;
2. choosing a fixed ANN (and/or Organism) which is trained on the Training Set. In this phase, the ANN learns to associate the input variables with those that are indicated as targets;
3. saving the weight matrix produced by the ANNs at the end of the training phase, and freezing it with all of the parameters used for the training;
4. showing the Testing Set to the ANN, so that in each case, the ANN can express an evaluation based on the training just performed. This procedure takes place for each input vector but every result (output vector) is not communicated to the ANN; in this way, the ANN is evaluated only in reference to the generalization ability that it has acquired during the Training phase;
5. constructing a new ANN with identical architecture to the previous one and repeating the procedure from point 1.

This general training plan has been further developed to increase the level of reliability of the generalization of the processing models. The experiments have been done using a random criterion of distribution of the samples. We have employed a cross-validation protocol with seven independent elaborations for every sample. It consists in dividing the sample seven times in 2 specular sub samples, containing each similar distribution of cases and controls.

## 3. Results

### *3.1. Demographic, clinical and neuropsychological data*

Ninety-four patients with FTD were recruited during eight consecutive months, at the Dementia Research Centers involved in the study [41 women; mean age 68.4 (SD = 8.4 years); mean education 8.5 years (SD = 4.5); MMSE = 23.6 adjusted score (SD = 2.9)] and 160 AD patients [102 women; mean age 77.7 (SD = 5.2 years); mean education 6.5 years (SD = 3.5); MMSE = 23.1 adjusted score (SD = 2.2)].

Patients with language disorders interfering with the ToL task were excluded when the adjusted score at the Token Test [35] was less than 29.

As expected, AD patients were significantly older ($p < 0.005$) and less educated ($p < 0.05$) than FTD patients. In the FTD group there were significantly more men ($p < 0.001$) and the duration of disease was longer ($p < 0.005$).

Table 1 shows main demographical and clinical features, as well as the AS, of the two groups.

Table 2 shows the values of TOL variables employed by ANNs: performance to each TOL problems (lond 1; lond 2... lond 12) plus total score (lond tot).

Table 1
Comparison of main demographic and neuropsychological data of the two groups of patients

| | AD | FTD |
|---|---|---|
| Number (M/F) | 160 (58/102) | 94 (53/41) |
| Age | 77.7 (SD 5.2) | 68.4 (SD 8.4) |
| Education | 6.5 (SD 3.4) | 8.5 (SD 4.5) |
| Duration of disease (months) | 23.2 (SD 15.8) | 32.5 (SD 21.9) |
| MMSE (adjusted scores) | 23.1 (SD 2.2) | 23.6 (SD 2.9) |
| Accuracy Score (adjusted scores) | 25.0(SD 7.3) | 23.1 (SD 7.8) |

Table 2
Comparison of TOL individual items in the two diagnosis group: (SD = standard deviation; C.I. = Confidence Interval)

| TOL items | FTD | | | AD | | | P |
|---|---|---|---|---|---|---|---|
| | mean | SD | C.I 95% | mean | SD | C.I 95% | value |
| *lond1* | 2.76 | 0.56 | 0.12 | 2.78 | 0.60 | 0.09 | N.S |
| *lond2* | 2.73 | 0.59 | 0.12 | 2.75 | 0.71 | 0.11 | N.S |
| *lond3* | 2.19 | 1.02 | 0.21 | 2.64 | 0.79 | 0.12 | N.S |
| *lond4* | 1.95 | 1.16 | 0.24 | 2.53 | 0.85 | 0.13 | N.S |
| *lond5* | 2.01 | 1.16 | 0.24 | 2.36 | 0.99 | 0.16 | N.S |
| *lond6* | 1.85 | 1.15 | 0.24 | 1.84 | 1.06 | 0.16 | N.S |
| *lond7* | 1.72 | 1.23 | 0.25 | 1.74 | 1.21 | 0.19 | N.S |
| *lond8* | 1.66 | 1.22 | 0.25 | 1.78 | 1.13 | 0.18 | N.S |
| *lond9* | 1.97 | 1.14 | 0.23 | 1.88 | 1.17 | 0.18 | N.S |
| *lond10* | 1.21 | 1.22 | 0.25 | 1.38 | 1.23 | 0.19 | N.S |
| *lond11* | 1.61 | 1.22 | 0.25 | 1.63 | 1.25 | 0.19 | N.S |
| *lond12* | 1.33 | 1.26 | 0.26 | 1.39 | 1.17 | 0.18 | N.S |
| *londtot* | 22.99 | 7.52 | 1.54 | 24.69 | 7.25 | 1.13 | N.S |

Table 3
Correlations between ToL AS score and tests examining global deterioration (MMSE) or executive functions in the two patients groups

| | AD r | P-value | FTD r | P-value |
|---|---|---|---|---|
| MMSE* [17] | 0.25037 | **0.0149** | 0.21866 | **0.0055** |
| CD* [21] | 0.32922 | **0.0015** | 0.24651 | **0.0017** |
| CDP* [21] | 0.22557 | **0.0316** | 0.27222 | **0.0005** |
| Numerical matrices* [18] | 0.22384 | **0.0301** | 0.31821 | $<$ **0.0001** |
| RCPM* [20] | 0.42060 | $<$ **0.0001** | 0.37393 | $<$ **0.0001** |
| Phonological fluency* [19] | 0.11912 | 0.2528 | 0.11397 | 0.1526 |
| Semantic fluency* [19] | 0.16774 | 0.1061 | 0.18856 | 0.0173 |

*Scores adjusted for age and education.

Table 4
Predictive values obtained in 7 independent testing sets

| **Neural network** | FDT | Alzheimer | Global accuracy |
|---|---|---|---|
| Back propagation (1) | 70.21 | 90 | 80.11 |
| Back propagation (2) | 68.09 | 90 | 79.04 |
| Back propagation (3) | 76.60 | 81.25 | 78.92 |
| Back propagation (4) | 70.21 | 88.75 | 79.48 |
| Back propagation (5) | 74.47 | 85 | 79.73 |
| Back propagation (6) | 70.21 | 91.25 | 80.73 |
| Back propagation (7) | 78.72 | 80 | 79.36 |
| **Average** | 72.64 | 86.61 | 79.63 |

### 3.2. Classical statistical analysis

AD performed better than FTD group for AS [25.03 (SD 7.3) vs 23.12 (SD 7.8); $p = 0.051$], however the ability of AS to discriminate FTD from AD was poor as evidenced with ROC (AUC 0.57). Among other neuropsychological tests, the phonological fluency score resulted better (AUC 0.69), namely for women (AUC 0.74).

Table 3 shows the correlations between AS, global deterioration as measured by MMSE and some neuropsychological tasks tapping executive abilities.

For both AD and FTD accuracy score correlated with RCPM and, at lesser extent, with attention/concentration (numerical matrices) in FTD and constructional praxia (CD) in AD.

When considering complexity score, FTD resulted more impaired than AD patients (−13.3% vs −1.5%, $p < 0.01$), when they had to solve problems with more then 3 moves instead of 2 or less.

### 3.3. Neural networks analysis

The Fig. 1 shows the value of correlation index between the variables of TOL (score of each of 12 problems and total score), gender, schooling and age and the FTD target. It is clear that age has the most relevant value ($r = 0.57$) followed by performance on the TOL problems 4 and 3 ($r = 0.28$ and 0.24 respectively). From an overall point of view the r values are anyway rather low (no correlation index higher than 0.3 with the exception of age) and this justify the use of non linear approach with artificial neural networks.

The predictive results obtained with artifical neural networks are shown in Table 4.

The global predictive accuracy obtained with standard ANNs ranged from 79.04% to 80.73% (average 79.63%).

The corresponding area under the ROC curves for each experiment and for the average results is shown in Fig. 2.

The Fig. 3 shows the distribution of input relevance of each variable considered in the neural network model during the training. As expected, the scoring of the variable doesn't follow the linear correlation distribution, excepted for age.

The input relevance is a parameter showing in arbitrary units the actual degree of importance in the trained model of each variable.

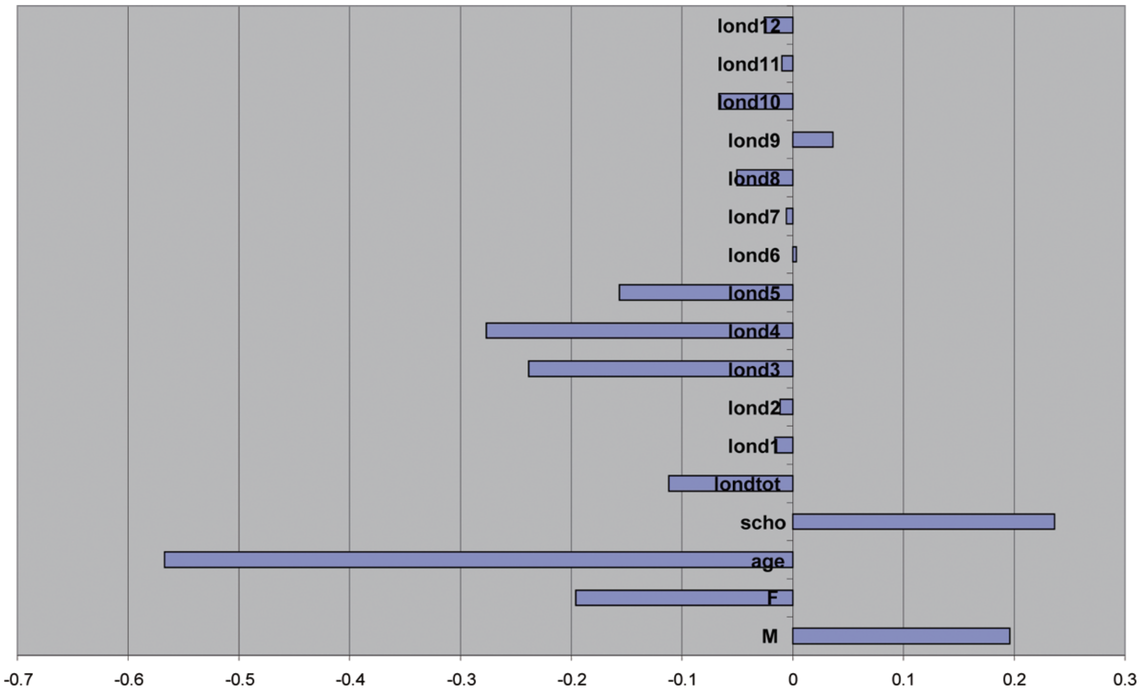

Fig. 1. Each bar represent the value of the correlation index R between the specific items of Tower of London test(lond 1; lond 2; etc.) total score of Tower of London test(lond tot) male and female gender, age, schooling years (scho) and presence of FTD. Negative value of R denote variables whose value is inversely correlated with the presence of FTD while positive value of R denote variables whose value is positively correlated with FTD presence.

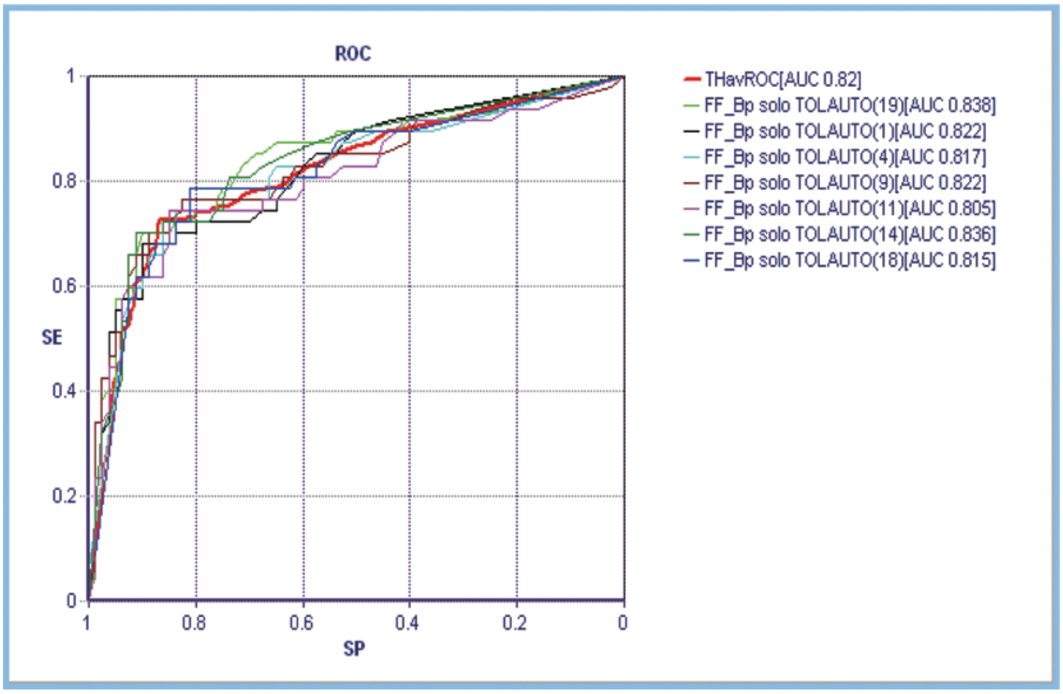

Fig. 2. ROC AUC of 7 testing experiments with ANNS.

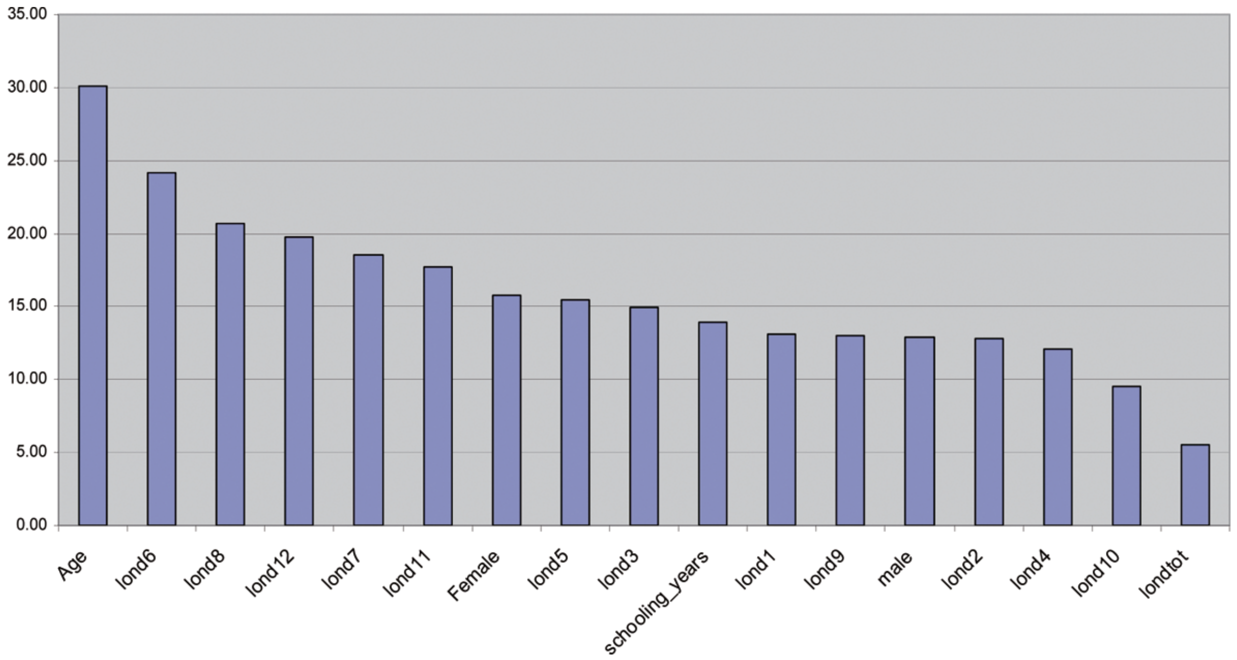

Fig. 3. Input relevance distribution of the variables in the neural network model.

## 4. Discussion

Despite a widespread use in experimental psychology, the exact nature of the cognitive processes involved in the execution of ToL remains elusive [40]. ToL measures the ability to plan and perform a complex visuospatial task with sufficient accuracy and without violating predefined rules [19,39]. Subjects, before moving the balls, need to plan the sequences of moves while reminding the previous ones. An impairment in ToL performance could occur either because of the inability to successfully inhibit inappropriate move selections at a specific point of the decisional pathway [1], or because of a deficit of visuospatial working memory, or a planning deficit [5]. Recent neuroimaging studies [33,41,42] have focused primarily on the role of the prefrontal dorsolateral and inferior parietal cortices during cognitive tasks involved in ToL which also induces functional activation of subcortical structures as caudate nucleus [36], striatum and precuneus [41].

In a previous paper [13] we reported that early AD patients were significantly impaired in visuospatial planning and problem solving as measured with ToL.

In the present paper, using classical statistical methods we were unable to accurately differentiate AD from FTD in single case study, whereas as a group, AD patients performed better in AS and CS. Based on neuropathological [36], behavioural [44] and imaging [17] data there is increased evidence of an early prefrontal dysfunction in AD, which could explain the occurrence of an early visuospatial planning deficit in this disease compared to the expected dysexecutive deficits found in FTD patients.

However, when Artificial Neural Networks' methodology was used to evaluate the Success Score (SS), the overall accuracy of this measure to discriminate AD from FTD becomes more accurate (79.6%).

In a clinical setting the AANs analysis of SS score on ToL reaches a diagnostic accuracy rarely obtained by other diagnostic tools, much more expensive and less patient-friendly, such as functional neuroimaging techniques [12,15].

To the best of our knowledge only three papers have been published focusing on the administration of ToL in patients with AD or FTD. Rainville et al. [30], using a different version of ToL, found significantly more rules breaking in AD patients, while controls made more moves to achieve the required position. As expected, AD patients were also significantly more impaired along with the complexity of the problem. The authors' comment was similar to the conclusions reported in our paper about the usefulness of this task in the clinical evaluation of AD patients [13].

In another study [7] comparing small samples of FTD and patients with focal frontal lobe lesions, demented were more impaired in both planning and execution than patients with focal lesions.

Marchegiani et al. [25] used the Krikorian's version of ToL to test 30 demented patients and 40 normal aged controls. As expected, demented made more errors on accuracy scores and showed longer execution time than controls. Moreover, Authors found a good correlation between MMSE and ToL scores and conclude that ToL provides complementary information to the MMSE and vice versa.

Only one previous study [40] tried to apply ToL in the differential diagnosis of AD vs FTD, along with several other neuropsychological and behavioural parameters. Using a computer version of ToL specifically devised for the study, they were able to test only 3 FTD patients vs 39 AD patients. As expected, ToL showed low capability to discriminate between FTD and AD.

A limitation of the present study relies on the fact that the rule violations were not recorded in our samples. Rule violations are difficult to define and to assess, although in previous papers with smaller size samples, they were similarly frequent in AD [30] and in FTD patients [7].

Another limitation of this study is the lack of any pathological or genetical confirmation of the clinical diagnoses. Even though mistakes are not uncommon in the clinical differentiation of AD from FTD, our sample was diagnosed by experienced professionals attending the Dementia Centers, routinely involved in the follow-up of the patients and consequently in the clinical confirmation of the diagnostic process.

Strengths of our study are the size of the sample studied and the accurate training of the neuropsychologists involved in the test administration.

Another interesting point resides on the suggestion to pursue alternative ways to the conventional statistical methodological approach, i.e. by using artificial neural networks analysis, which seems to increase the diagnostic accuracy between different types of dementia.

The comparison of results obtained with two these different statistical approaches, points out the need to employ systems really able to handle the disease complexity, instead of treating the data with reductionistic approaches that are unable to detect multiple interactions among variables.

Moreover, artificial neural networks, at variance with the classical statistical tests, can manage complexity even in the presence of small samples and to the subsequent unbalanced ratio between variables and records. Taking into account this connection, it is important to note that adaptive learning algorithms of inference, based on the principle of a functional estimation like artificial neural networks, overcome the problem of dimensionality.

In conclusion the simplified version of ToL was found an easy, fast to administer and user-friendly test to be included in the neuropsychological battery for the early diagnosis of AD vs FTD also in a single case study, along with a simple software dedicated to unconventional statistical methods, able to consider test complexity and to solve non-linear problems related to diagnostic or prognostic queries.

## References

[1] H. Amieva, L.H. Phillips, S. Della Sala and J.D. Henry, Inhibitory functioning in Alzheimer's disease, *Brain* **127** (2004), 949–996.

[2] Y. Anzai and H. Simon, The theory of learning by doing, *Psychology Revue* **86** (1979), 124–131.

[3] A. Basso, E. Capitani and M. Laiacona, Raven's Coloured Progressive Matrices: normative values on 305 adult normal controls, *Functional Neurology* **2** (1987), 189–194.

[4] M. Buscema, Special issue on artificial neural networks and complex social systems, I. Theory, *Substance Use & Misuse* **33** (1998), 1–220.

[5] H.P. Carder, S.J. Handley and T.J. Perfect, Deconstructing the Tower of London: alternative moves and conflict resolution as predictors of task performance, *Quarterly Journal of Experimental Psychology* **57A**(8) (2004), 1459–1483.

[6] G.A. Carlesimo, C. Caltagirone and G. Gainotti, Batteria per la valutazione del Deterioramento Mentale (Parte II): standardizzazione e affidabilità diagnostica nell'identificazione di pazienti affetti da sindrome demenziale, *Archivi di Psicologia, Neurologia e Psichiatria* **4** (1995), 461–470.

[7] D. Carlin, J. Bonerba, M. Phipps, G. Alexander, M. Shapiro and J. Grafman, Planning impairments in frontal lobe dementia and frontal lobe lesion patients, *Neuropsychologia* **38**(5) (2000), 655–666.

[8] S.S. Cross, R.F. Harrison and R.I. Kennedy, Introduction to neural networks, *Lancet* **346** (1995), 1075–1079.

[9] J.E. Dayhoff and J.M. DeLeo, Artificial Neural Networks. Opening the black box, *Cancer* **91**(S9) (2001), 1615–1635.

[10] M.B. Denkla, A theory and model of executive function: a neuropsychological perspective. in: *Attention, Memory and Executive Functions*, G.R. Krasnegor and N.A. Krasnegor, eds, Baltimore, Brookes Pub, 1996, pp. 263–278.

[11] M. Di Luca, E. Grossi, B. Borroni, M. Zimmermann, E. Marcello, F. Colciaghi, F. Gardoni, M. Intraligi, A. Padovani and M. Buscema, Artificial neural networks allow the use of simultaneous measurements of Alzheimer disease markers for early detection of the disease, *Journal of Translational Medicine* **3** (2005), 30.

[12] N.L. Foster, J.L. Heidebrink, C.M. Clark, W.J. Jagust, S.E. Arnold, N.R. Barbas, C.S. De Carli, R.S. Turner, R.A. Koeppe, R. Higdon and S. Minoshima, PET-FDG improves accuracy in distinguishing frontotemporal dementia and Alzheimer's disease, *Brain* **130** (2007), 2616–2635.

[13] M. Franceschi, P. Caffarra, L. De Vreese, O. Pelati, S. Pradelli, R. Savarè, R. Cerutti and E. Grossi, Visuospatial planning and problem solving in Alzheimer's disease patients: a study with the Tower of London, *Dementia and Geriatric Cognitive Disorders* 24 (2007), 424–428.

[14] C.A. Gregory, M. Orrell, B. Sahakian and J.R. Hodges, Can frontotemporal dementia and Alzheimer's disease be differentiated using a brief battery of tests? *Internation Journal of Geriatric Psychiatry* **12** (1997), 375–383.

[15] E. Kapaki, G.P. Paraskevas, S.G. Papageorgiou, A. Bonakis, N. Kalfakis, I. Zalonis and D. Vassilopoulos, Diagnostic value of CSF biomarker profile in frontotemporal lobar degeneration, *Alzheimer Disease and Associated Disorders* **22** (2008), 47–53.

[16] A. Kertesz, W. Davidson, P. Mc Cabe and D. Munoz, Behavioral quantitation is more sensitive than cognitive testing in frontotemporal dementia, *Alzheimer Disease and Associated Disorders* **17** (2003), 223–229.

[17] W.E. Klunk, H. Engler, A. Nordberg, Y. Wang, G. Blomqvist, D.P. Holt, M. Bergstrom, I. Savitcheva, G.F. Huang, S. Estrada, B. Ausen, M.L. Debnath, J. Barletta, J.C. Price, J. Sandell, B.J. Lopresti, A. Wall, P. Koivisto, G. Antoni, C.A. Mathis and B. Langstrom, Imaging brain amyloid in Alzheimer's disease with Pittsburgh Compound-B, *Annals of Neurology* **55** (2004), 306–319.

[18] D.S. Knopman, R.C. Petersen, S.D. Edland, R.H. Cha and W.A. Rocca, The incidence of frontotemporal lobar degeneration in Rochester, Minnesota, 1990 through 1994, *Neurology* **58** (2004), 1615–1621.

[19] R. Krikorian, J. Bartok and N. Gay, Tower of London procedure: a standard method and developmental data, *Journal of Clinical and Experimental Neuropsychology* **16**(6) (1994), 840–850.

[20] D.J. Libon, S.X. Xie, P. Moore, J. Farmer, S. Antani, G. Mc Cawley, K. Cross and M. Grossman, Patterns of neuropsychological impairment in frontotemporal dementia, *Neurology* **68** (2007), 369–375.

[21] F. Licastro, E. Porcellini, M. Chiappelli, P. Forti, M. Buscema, G. Ravaglia and E. Grossi, Multivariable network associated with cognitive decline and dementia, *Neurobiology of Aging*, e-pub online May 15 2008.

[22] R.M. Liscic, M. Storandt, N.J. Cairns and J.C. Morris, Clinical and psychometric distinction of frontotemporal and Alzheimer dementias, *Archives of Neurology* **64** (2007), 535–540.

[23] G. McKhann, D. Drachman, M. Folstein, R. Katzman, D. Price and E.M. Stadlan, Clinical diagnosis of Alzheimer's disease: report of the NINCDS/ADRDA Work Group under the auspices of Department of Health and Human Services Task Force on Alzheimer's disease, *Neurology* **34** (1984), 939–944.

[24] E. Magni, G. Binetti, A. Bianchetti, R. Rozzini and M. Trabucchi, Mini-Mental State Examination: a normative study in Italian elderly population, *European Journal of Neurology* **3** (1996), 1–5.

[25] A. Marchegiani, M.V. Giannelli and P.R. Odetti, The Tower of London test: a test for dementia, *Aging Mental Health* **23** (2009), 1–4.

[26] D. Neary, J.S. Snowden, L. Gustafson, U. Passant, D. Stuss, S. Black, M. Freedman, A. Kertesz, P.H. Rober, M. Albert, K. Boone, B.L. Miller, J. Cummings and D.F. Benson, Frontotemporal lobar degeneration: a consensus on clinical diagnostic criteria, *Neurology* **51** (1998), 1546–1554.

[27] G. Novelli, C. Papagno and E. Capitani, Test di fluenza verbale, *Archivi di Psicologia, Neurologia e Psichiatria* **47** (1986), 477–506.

[28] R.J. Perry and J.R. Hodges, Differentiating frontal and temporal variant of frontotemporal dementia from Alzheimer's disease, *Neurology* **54** (2000), 2277–2841.

[29] G.D. Rabinovici, W.W. Seeley, E.J. Kim, M.L. Gorno-Tempini, K. Rascovsky, T.A. Pagliaro, S.C. Allison, C. Halabi, J.H. Kramer, J.K. Johnson, M.W. Weiner, M.S. Forman, J.Q. Trojanowski, S.J. Dearmond, B.L. Miller and H.J. Rosen, Distinct MRI atrophy patterns in autopsy-proven Alzheimer's disease and frontotemporal lobar degeneration, *American Journal of Alzheimer's Disease and Other Dementias* **22**(6) (2007), 474–488.

[30] C. Rainville, H. Amieva, S. Lafont, J.F. Dartigues, J.M. Orgogozo and C. Fabrigoule, Executive function deficits in patients with dementia of the Alzheimer's type: a study with a Tower of London task, *Archives of Clinical Neuropsychology* **17**(6) (2002), 513–530.

[31] P.M. Rossini, M. Buscema, M. Capriotti, E. Grossi, G. Rodriguez, C. Del Percio and C. Babiloni, Is it possible to automatically distinguish resting EEG data of normal elderly vs. mild cognitive impairment subjects with high degree of accuracy? *Clinical Neurophysiology* **119**(7) (2008), 1534–1545.

[32] T.A. Salthouse, Relationship between cognitive abilities and measures of executive functioning, *Neuropsychology* **19**(4) (2005), 532–545.

[33] U. Shall, P. Johnston, J. Lagopoulos, M. Juptner, W. Jentzen, R. Thienel, A. Dittmann-Balcar, S. Bender and P.B. Ward, Functional brain maps of Tower of London performance: a positron emission tomography and functional magnetic resonance imaging study, *Neuroimage* **20** (2003), 1154–1161.

[34] J.I. Sheikh and J.A. Yesavage, Geriatric Depression Scale (GDS): Recent evidence and development of a shorter form, *Clinical Gerontology* **5** (1986), 166–173.

[35] H. Spinnler and G. Tognoni, *Standardizzazione e Taratura Italiana di Test Neuropsicologici*, in Italian Journal of Neurological Sciences, Masson Italia Periodici, Milano 1987.

[36] D.R. Thal, U. Rub, M. Orantes and H. Braak, Phases of A-beta deposition in the human brain and its relevance for the development of AD, *Neurology* **58** (2002), 1791–1800.

[37] J.C. Thompson, C.L. Stapford, J.S. Snowden and D. Neary, Qualitative neuropsychological performance characteristics in frontotemporal dementia and Alzheimer's disease, *Journal of Neurology, Neurosurgery and Psychiatry* **76** (2005), 920–927.

[38] J.M. Unterrainer, B. Rahm, C.P. Kaller, R. Leonhart, K. Quiske, K. Hoppe-Seyler, C. Meier, C. Muller and U. Halsband, Planning abilities and the Tower of London: is this task measuring a discrete cognitive function? *Journal of Clinical and Experimental Neuropsychology* **26** (2004), 846–856.

[39] J.M. Unterrainer, B. Rahm, U. Halsband and C.P. Kaller, What is in a name: comparing the Tower of London with one of its variants, *Cognitive Brain Research* **25** (2005), 418–428.

[40] A.H. Valverde, A. Jimenez-Escrig, J. Gobernado and M. Baron, A short neuropsychologic and cognitive evaluation of frontotemporal dementia, *Clinical Neurology and Neurosurgery* **111** (2009), 251–255.

[41] A.O. Van den Heuvel, H.J. Groenewegen, F. Barkhof, R.H.C. Lazeron, R. van Dyck and D.J. Veltman, Frontostriatal system in planning complexity: a parametric functional megnetic rersonance version of Tower of London, *Neuroimage* **18** (2003), 367–374.

[42] G. Wagner, K. Koch, J.R. Reichenbach, H. Sauer and R.J.M. Schlosser, The special involvement of the rostrolateral prefrontal cortex in planning abilities: an event-related fMRI study with the Tower of London, *Neuropsychologia* **44**(12) (2006), 2337–2347.

[43] J.H. Wasson, H.C. Sox, R.K. Neff and L. Goldman, Clinical prediction rules: Applications and methodological standards, *New England Journal of Medicine* **313** (1995), 793–799.

[44] S.L. Willis, R. Allen-Burge, M.M. Dolan, R.M. Bertrand, J. Yesavage and J.L. Taylor, Everyday problem solving among individuals with Alzheimer's disease, *Gerontologist* **38**(5) (1998), 569–577.

[45] J.C. Wyatt, Decision support systems, *Journal of Royal Society of Medicine* **93** (2000), 629–633.
